# A seed mixture increases dominance of resistance to Bt cotton in *Helicoverpa zea*

**DOI:** 10.1038/srep09807

**Published:** 2015-05-07

**Authors:** Thierry Brévault, Bruce E. Tabashnik, Yves Carrière

**Affiliations:** 1 Centre de Coopération Internationale en Recherche Agronomique pour le Développement (CIRAD), UPR 115 AIDA, F-34398 Montpellier, France; 2 Department of Entomology, University of Arizona, Tucson, AZ 85721, USA

## Abstract

Widely grown transgenic crops producing insecticidal proteins from *Bacillus thuringiensis* (Bt) can benefit agriculture, but adaptation by pests threatens their continued success. Refuges of host plants that do not make Bt toxins can promote survival of susceptible insects and delay evolution of resistance, particularly if resistance is inherited as a recessive trait. However, data have been lacking to compare the dominance of resistance when Bt and non-Bt seeds are planted in random mixtures versus separate blocks. Here we report results from greenhouse experiments with transgenic cotton producing Bt toxin Cry1Ac and the bollworm, *Helicoverpa zea*, showing that the dominance of resistance was significantly higher in a seed mixture relative to a block of Bt cotton. The proportion of larvae on non-Bt cotton plants in the seed mixture was also significantly higher than expected under the null hypothesis of random distribution. In simulations based on observed survival, resistance evolved 2- to 4.5-fold faster in the seed mixture relative to separate blocks of Bt and non-Bt cotton. These findings support previous modelling results indicating that block refuges may be more effective than seed mixtures for delaying resistance in pests with mobile larvae and inherently low susceptibility to the toxins in Bt crops.

Genetically engineered crops producing insecticidal proteins from *Bacillus thuringiensis* (Bt) were planted on 76 million hectares worldwide in 2013, including 75% of the corn and 76% of the cotton in the United States^1^,^2^. These Bt crops can provide several benefits, including reduced reliance on chemical insecticides, conservation of natural enemies, regional pest suppression, and increased or less variable yields^3^. Because rapid adaptation of insect pests to Bt crops threatens the sustainability of these benefits, the refuge strategy has been adopted widely to delay evolution of resistance^3^,^4^. The basic idea underlying this strategy is that the rare resistant individuals surviving on Bt crops mate primarily with the relatively abundant susceptible individuals from refuges of non-Bt host plants^4^,^5^,^6^. This strategy is particularly effective for delaying resistance that is inherited as a functionally recessive trait, because the heterozygous progeny produced by matings between resistant and susceptible adults do not survive on the Bt crops. Conversely, if inheritance of resistance is dominant, the progeny from matings between resistant and susceptible adults survive on Bt crops, and refuges are less effective for delaying resistance.

Although a substantial body of theory and data show that refuges can delay insect adaptation to Bt crops^4^,^5^,^6^,^7^,^8^,^9^,^10^, the optimal spatial scale for planting refuges remains unresolved. From the initial commercialization of Bt crops in 1996 until 2010, regulations in the United States mandated refuges of non-Bt plants in blocks, either in separate fields, rows, or strips^11^. In 2010, the regulations were modified to include refuges planted with mixtures of Bt and non-Bt seeds that yield a random array of Bt and non-Bt plants side-by-side within fields^12^.

Seed mixtures have several advantages relative to block refuges, including elimination of the problem of farmers who do not comply with block refuge requirements^13^. If larvae move between plants, however, modeling results suggest that seed mixtures could accelerate resistance evolution by increasing the dominance of resistance or by reducing survival of susceptible insects and thereby decreasing the effective size of refuges^14^,^15^,^16^,^17^. Mallet and Porter^14^ proposed that when individual larvae feed on both Bt and non-Bt plants, seed mixtures can increase the dominance of resistance by increasing survival of heterozygous larvae relative to homozygous susceptible larvae. Despite potentially profound implications for the durability of Bt crops, this hypothesis has not been tested previously.

Here we used greenhouse experiments with a model system to compare the dominance of resistance to transgenic cotton producing Bt toxin Cry1Ac grown in seed mixtures versus blocks in the bollworm, *Helicoverpa zea*. Cry1Ac is the sole Bt toxin made by the first type of Bt cotton, which was grown in the United States from 1996 to 2010 and is still planted on millions of hectares in some other countries including China^1^,^18^. Previous results show that *H. zea* has relatively low inherent susceptibility to Cry1Ac^19^ and non-recessive inheritance of resistance to Cry1Ac cotton plants grown in blocks^18^. Significantly decreased susceptibility to Cry1Ac in some field-selected populations of this pest in the United States provided the first evidence of field-evolved resistance to a Bt toxin produced by a transgenic crop^4^,^20^,^21^,^22^. The results here show the dominance of *H. zea* resistance to Bt cotton producing Cry1Ac was significantly higher for a seed mixture than a block. Incorporation of these data into a population genetic model suggest that the observed increase in dominance could accelerate evolution of resistance by 2- to 4.5-fold.

## Results

We compared the dominance of resistance to Bt cotton producing Cry1Ac (referred to hereafter as Bt cotton) planted in a block of 100% Bt cotton versus a seed mixture with a random array of 78% Bt cotton and 22% non-Bt cotton. We tested three types of *H. zea*: a field-derived strain from Georgia (GA) that was exposed to Bt toxins only in the field, a resistant strain (GA-R) that was derived from GA and selected in the laboratory for resistance to Cry1Ac, and the F1 progeny from reciprocal crosses between these strains. We used the survival of these three types of *H. zea* on plants in the greenhouse to calculate the dominance parameter *h*, which varies from 0 for completely recessive resistance to 1 for completely dominant resistance^23^.

**Figure 1:**
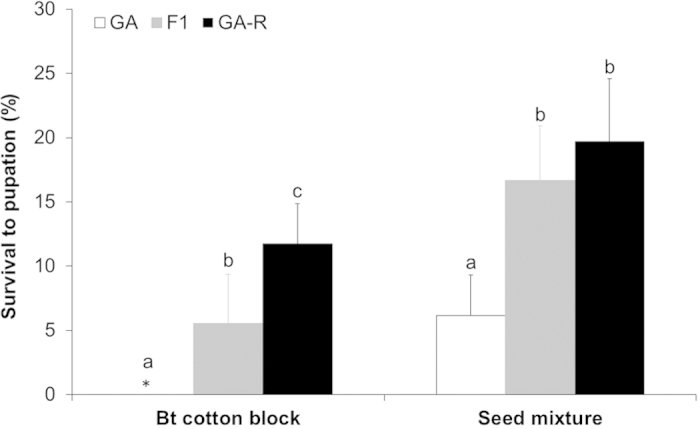
Survival to pupation (+95% CI) in arrays of 100% Bt cotton and seed mixtures of 78% Bt cotton and 22% non-Bt cotton in *H. zea* from a field-derived strain (GA), a resistant strain (GA-R), and their F1 progeny. The asterisk indicates 0% survival of GA in arrays of 100% Bt cotton. On Bt cotton and the seed mixture, different letters indicate significant differences between the strains (linear contrasts, *P*<0.05).

Inheritance of resistance to Bt cotton was not recessive for either the seed mixture (*h *= 0.76, SE* *= 0.12) or the block (*h *= 0.48, SE* *= 0.12). Moreover, the dominance of resistance to Bt cotton was significantly higher in the seed mixture than in the block (F_1, 7_* *= 5.7, *P *= 0.048, Table S1). In both the seed mixture and the Bt cotton block, survival varied significantly among the three insect types and was significantly higher for F1 than GA (*P *< 0.01 in each linear contrast, Fig. 1, Table S2). Survival was also significantly higher for GA-R than F1 in the Bt cotton block (*P *= 0.043), but it did not differ significantly between GA-R and F1 in the seed mixture (*P *= 0.47) (Fig. 1, Table S2). The mean increase in survival for the seed mixture relative to the Bt cotton block was significantly higher for F1 (11%, SE* *= 2.3%) than GA (6.2%, SE* *= 2.0%) (paired t-test, t_8_* *= 2.87, *P *= 0.02), but did not differ between F1 and GA-R (8.0%, SE* *= 2.1%) (paired t-test, t_8_* *= 1.25, *P *= 0.25).

**Figure 2:**
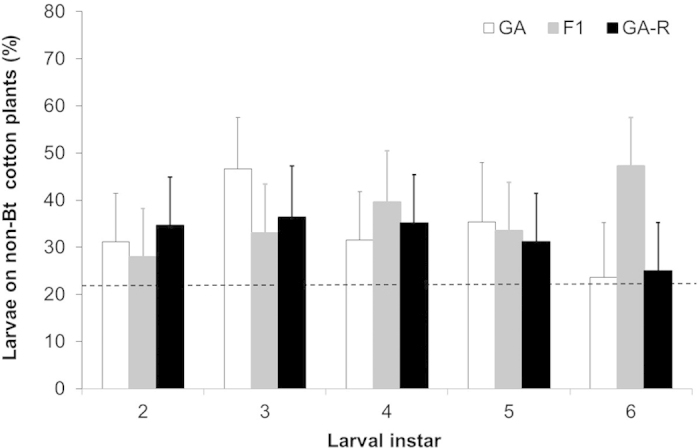
Percentage of *H. zea* larvae on non-Bt cotton plants (+SE) in a seed mixture of 78% Bt cotton and 22% non-Bt cotton for a field-derived strain (GA), a resistant strain (GA-R), and their F1 progeny. The dashed line represents expected 22% of larvae on non-Bt plants if larvae occur on Bt and non-Bt plants in proportion of their relative abundance.

In the seed mixture, the percentage of larvae on non-Bt cotton was significantly higher than the expected 22% based on the relative abundance of non-Bt cotton plants (Fig. 2) (mean* *= 33%, 95% ci* *= 26–41%; t_26_* *= 3.2, *P *= 0.0046). The percentage of larvae on non-Bt cotton plants was not affected significantly by insect type, instar, or the interaction between these two factors (ANOVA, *P* > 0.72 for each effect) (Supplementary Table 3). Nevertheless, F1 larvae occurred significantly more often on non-Bt plants than the expected 22% (least squares mean* *= 36%, 95% ci* *= 24–48%), while GA and GA-R larvae did not (lsm* *= 32%, 95% ci* *= 20–45% and lsm* *= 32%, 95% ci* *= 21–44%, respectively).

In simulations of population genetic models incorporating the observed survival of the three types of *H. zea* in the seed mixture and Bt cotton block, resistance evolved faster with a seed mixture than with a block refuge (Table 1). A sensitivity analysis shows that, as survival of susceptible insects on blocks of non-Bt cotton increased from 35 to 95%, the rate of evolution of resistance in the seed mixture relative to blocks of Bt and non-Bt cotton increased from 2-fold to 4.5-fold (Table 1).

## Discussion

The results here with *H. zea* tested on Bt and non-Bt cotton plants in the greenhouse provide the first experimental evidence that the dominance of pest resistance to a Bt crop was higher in a seed mixture than in a block of the Bt crop. The results also show that, as hypothesized by Mallet and Porter^14^, the higher dominance in the seed mixture occurred because the increase in survival on the seed mixture relative to the block of Bt cotton was greater for putative heterozygotes (F1) than for susceptible insects (GA). We also found that in a seed mixture, the percentage of larvae on non-Bt cotton was significantly higher than expected based on the relative abundance of Bt and non-Bt cotton plants. This difference was significant for F1 larvae considered alone, but not for larvae from GA and GA-R considered separately.

Simulations based on the observed survival on cotton plants of the three insect types (GA, GA-R and F1) show that the projected rate of resistance evolution is 2 to 4.5 times higher in seed mixtures relative to blocks of Bt and non-Bt cotton. The experimental evidence and simulation results reported here support previous modelling results^14^,^17^ indicating that seed mixtures may be less effective than block refuges for managing resistance to Bt crops in pests such as *H. zea* in which larvae commonly move between plants.

The lack of survival of larvae from GA on blocks of Bt cotton indicates that resistance alleles were rare in this field-derived strain. Although we cannot rule out the possibility that the presence of some resistance alleles in GA might have affected the dominance of resistance, we show that such resistance alleles cause an underestimation of the true increase in dominance in the seed mixture relative to block of Bt cotton (Supplementary Methods and Table S4). This occurs because dominance is underestimated in seed mixtures and overestimated in blocks of Bt cotton when resistance alleles occur in GA. Accordingly, our estimate of the increase in dominance in the seed mixture relative to block of Bt cotton is conservative.

In addition to larval movement between plants, inherently low susceptibility to the toxins in Bt crops is expected to increase the chances that dominance of resistance is higher in seed mixtures than blocks of Bt crops. For example, compared with two other major pests of cotton in the United States, *Pectinophora gossypiella* and *Heliothis virescens*, *H. zea* has much lower inherent susceptibility to Cry1Ac^19^. This inherently low susceptibility of *H. zea* to Cry1Ac is reflected in non-recessive resistance of this pest to blocks of Bt cotton that produces Cry1Ac, as reported previously^18^ and confirmed in this study. Because inheritance of resistance of *H. zea* to Cry1Ac cotton has intermediate dominance when the Bt cotton is grown in blocks (*h *= 0.48 in this study), even small increases in the survival of heterozygotes relative to homozygous susceptible insects can increase dominance.

Previously published experimental results testing the idea that providing larvae the opportunity to feed on both Bt and non-Bt plant tissues increases the dominance of resistance to a commercial Bt crop are available only for *P. gossypiella* and Bt cotton producing Cry1Ac^24^. The larvae of this pest eat cotton seeds and require only a single cotton boll to complete development. Pollen-mediated gene flow between Bt and non-Bt cotton plants yields bolls with various proportions of Bt and non-Bt seeds^24^,^25^. Although *P. gossypiella* larvae are unlikely to move between plants, feeding on Bt and non-Bt seeds within such bolls could increase dominance in a manner analogous to what has been proposed for mixtures of Bt and non-Bt plants within fields. However, the dominance of resistance did not increase significantly in experiments comparing survival of Cry1Ac-susceptible, heterozygous, and Cry1Ac-resistant larvae in artificial bolls with different mixtures of Bt and non-Bt seeds^24^.

In a model system with *Plutella xylostella* and non-commercial Bt broccoli producing Cry1Ac, larvae in seed mixtures in the greenhouse rarely moved from non-Bt plants to Bt plants, and all F1 larvae that moved from Bt to non-Bt plants died^26^. These results imply that larval movement between Bt and non-Bt plants in this system would be unlikely to increase the dominance of resistance. Similarly, in a selection experiment conducted in the field using the same model system, the percentage of larvae susceptible to Cry1Ac at the end of the experiment was not lower in seed mixture plots (98%) compared with plots containing separate blocks of Bt and non-Bt plants (97%), which indicates seed mixtures did not accelerate evolution of resistance^27^. In contrast with the two cases summarized above, which both involve pests with high inherent susceptibility to Cry1Ac and completely recessive inheritance of resistance to Bt plants producing Cry1Ac (*h *= 0)^28^,^29^,^30^, we found here that seed mixtures significantly increased dominance of resistance in *H. zea*, which has mobile larvae and inherently low susceptibility to Cry1Ac^6^,^18^,^19^.

The experiments in this study tested Bt cotton plants producing a single Bt toxin that are still used in many countries other than the United States and Australia^1^,^4^,^18^, but multi-toxin Bt crops are becoming increasingly common, particularly “pyramids” producing two or more toxins that kill the same insect^3^,^4^,^18^,^31^. In the United States, Bt cotton producing only Cry1Ac is no longer registered and seed mixtures are registered for pyramided Bt corn, but not for Bt cotton^13^. Therefore, the results from the model system studied here cannot be extrapolated directly to current field conditions in the United States.

Nonetheless, the assumptions of the pyramid strategy are often not met in pests such as *H. zea* with low inherent susceptibility to Bt toxins^4^,^6^,^18^,^31^,^32^, and the ability of seed mixtures of pyramided crops to delay resistance in such pests remains unclear. Even with no larval movement between corn plants, pollen-mediated gene flow could substantially accelerate evolution of resistance in seed mixtures relative to block refuges for insects that eat corn kernels. Gene flow between Bt and non-Bt corn in seed mixtures is extensive and produces a mosaic of Bt and non-Bt kernels in ears of non-Bt corn plants^33^,^34^,^35^,^36^. In pests such as *H. zea* and *Helicoverpa armigera* that eat kernels, the Bt toxins in kernels of refuge plants in seed mixtures could speed resistance by killing susceptible larvae and reducing effective refuge size^36^,^37^, increasing dominance of resistance, or both. Empirical data are urgently needed to evaluate the capacity of seed mixtures of pyramided plants to delay resistance.

## Methods

### Insect strains and rearing

We used a field-derived strain of *H. zea* that was reared without exposure to Bt toxins (GA), a resistant strain derived from GA that was selected in the laboratory with Cry1Ac for nine generations (GA-R)^18^, and the F1 progeny from reciprocal crosses between these strains (see Supplementary Methods 1). The GA strain, which was started with 180 larvae collected from Bt corn in Georgia, initially had 55-fold resistance to Cry1Ac in diet bioassays relative to a susceptible laboratory strain^18^. The GA-R strain was started with insects from the GA strain and selected in the laboratory for resistance to Cry1Ac. Before the current study, GA-R had been selected with increasing concentrations of Cry1Ac in diet for nine non-consecutive generations and had 560-fold resistance to Cry1Ac relative to a laboratory susceptible strain^18^. Because GA and GA-R share a similar rearing history and genetic background, any differences in performance on non-Bt and Bt cotton among GA, GA-R and their F1 progeny are likely to be caused by one or more genes that confer resistance to Bt toxins.

### Greenhouse experiments

We used Bt cotton producing Cry1Ac (DP 448 B, referred to hereafter as Bt cotton) and non-Bt cotton (DP 5415). We measured the survival and distribution of *H. zea* larvae in the greenhouse with sets of nine plants in each of two types of arrays: (i) 100% Bt cotton (nine Bt cotton plants) and (ii) seed mixture with seven Bt cotton plants (78%) and two non-Bt cotton plants (22%) (Fig. S1). *H. zea* females do not discriminate for oviposition between Bt and non-Bt plants^37^. Eighteen neonates (<24 h old) from one of the three insect types (GA, GA-R and F1 progeny) were individually transferred on terminal leaves of each type of array (two larvae per plant). In each of three successive temporal replicates, the combination of insect type and plant array type (n* *= 6) was randomized within each of three spatial replicates, yielding a total of nine replicates for each insect type on Bt cotton block or seed mixture (Fig. S2).

We monitored larval instar and location (Bt cotton or non-Bt cotton) every three days after we put the neonates on plants. Late 6^th^ instars were transferred individually to 470 mL clear plastic cups containing the boll on which larvae were found and the closest boll from the same plant. Cups were inspected every 3 days until pupation.

For each of the three temporal replicates, plant structures (i.e., leaves, squares, pink flowers, bolls) from each of three randomly selected non-Bt cotton plants and six Bt cotton plants were collected the day before transfer of neonates to arrays. ELISA tests (Quantiplate kit, Envirologix) revealed that 100% and 0% of the plant structures from Bt and non-Bt cotton plants produced Cry1Ac, respectively.

### Dominance of resistance

Dominance of resistance (*h*) is typically used to measure fitness of heterozygous individuals (*rs*) relative to homozygous susceptible (*ss*) and homozygous resistant (*rr*) individuals exposed to a single Bt toxin or Bt crop^18^,^23^. Dominance is calculated as: *h *= (W_rs_ – W_ss_)/(W_rr_ – W_ss_), where W_ss_, W_rs_, and W_rr_ refer to fitness of *ss*, *rs* and *rr*, respectively. Values of *h* vary from 0 for completely recessive resistance (fitness of *rs *= fitness of *ss*) to 1 for completely dominant resistance (fitness of *rs *= fitness of *rr*)^18^,^23^. As in Heuberger *et al.*^24^ where *h* was estimated in *P. gossypiella* larvae feeding in bolls with various proportions of Bt and non-Bt seeds, here we used *h* to measure dominance in arrays of plants (seed mixture or Bt cotton block) as: *h *= (W_F1_ – W_GA_)/(W_GA-R_ – W_GA_), where W_GA_, W_F1_ and W_GA-R_ are the survival from neonate to pupae of GA, F_1_, and GA-R, respectively. Although it is reasonable to assume that GA-R was fixed for resistance alleles, GA was probably not fixed for alleles conferring susceptibility. The presence of resistance alleles in GA, and calculation of *h* based on observed survival of GA, F1 and GA-R, causes underestimation of the increase in *h* in the seed mixture relative to the block of Bt cotton (see Supplementary Methods and Discussion).

### Data analysis

We used three-way ANOVA to evaluate effects on dominance (*h*) of plant configuration (seed mixture vs. Bt cotton block), temporal replicate, and spatial replicate nested within temporal replicate. We compared survival to pupation of GA, GA-R, and F1 in the seed mixture and Bt cotton block using logistic regression for binomial counts followed by linear contrasts. Explanatory variables were temporal replicate, spatial replicate nested within temporal replicate, and insect type (GA, GA-R, or F1). It was previously hypothesized that the higher dominance in seed mixture vs. block of Bt crop occurs due to a higher increase in survival on seed mixture relative to block of Bt crop in heterozygotes than susceptible insects. To test this hypothesis, we used paired t-tests to compare the increase in percentage survival in the seed mixture relative to the block of Bt cotton in F1 vs. GA and F1 vs. GA-R (nine values of change in percentage survival were calculated for each insect type and temporal replicate).

The expected percentage of larvae on non-Bt cotton in seed mixture arrays is 22% if larvae occur on Bt and non-Bt plants in proportion to the relative abundance of the plants. We used one-sample t-test to test whether the percentage of larvae on non-Bt cotton plants exceeded 22%. To account for the potential lack of independence of the position of larvae of successive instars, the response variable was the percentage of larvae observed on non-Bt plants in each array, calculated from the sum of larvae of second to sixth instar observed on Bt and non-Bt plants (see details in Supplementary Methods 1).

For each insect type in a particular seed mixture array, data were pooled across observation periods to determine the percentage of larvae of each instar on non-Bt cotton. We used ANOVA to assess whether the insect types differed in their occurrence on non-Bt cotton plants in seed mixtures. The response variable was the percentage of larvae on non-Bt cotton, and explanatory variables were temporal replicate, spatial replicate nested within temporal replicate, array nested within temporal and spatial replicate (arrays were experimental units measured several times), strain, instar, and the interaction between strain and instar. We considered overlap of the 95% confidence interval with 22% to evaluate whether the percentage of larvae of each type on non-Bt cotton plants exceeded 22%. All results reported from ANOVA or logistic regression incorporate effects of spatial and temporal replicates and array (see details in Supplementary Methods 1). Statistical analyses were performed in JMP 9.0 (SAS Institute, Cary, NC).

### Population genetic model

We used a deterministic population genetic model^16^ in which resistance was conferred by a single locus with two alleles: *r* conferring resistance and *s* susceptibility to Bt cotton. We assumed random mating and initial *r* allele frequency of 0.001. The time to resistance was the number of generations until the *r* allele frequency exceeded 0.50 Fitness of each genotype was based on the observed survival from neonate to pupation in the greenhouse experiment: 6% for GA, 17% for F1, and 20% for GA-R in the seed mixture; and 0% for GA, 6% for F1, and 12% for GA-R in blocks of Bt cotton (Fig. 1). Previously reported results from an experiment conducted simultaneously with this study show that survival on non-Bt cotton (DP 5415) was 71% in GA, 62% in GA-R and 66% in the F1 progeny, with mean survival of 66% (Fig. 2 of ref. 18). No significant difference in survival on non-Bt cotton occurred between GA and GA-R (χ2* *= 0.97, df* *= 1, P* *= 0.33, ref. 18) or between GA and F1 (χ2* *= 0.38, df* *= 1, P* *= 0.54). Thus, we assumed that survival on blocks of non-Bt cotton did not vary among genotypes. We used a sensitivity analysis to examine effects on evolution of resistance of three values of survival on blocks of non-Bt cotton: 35, 65 and 95%.

## Author Contributions

T.B. and Y.C. designed the study. T.B. performed the experiments. T.B., Y.C., and B.E.T. analysed the data. All authors discussed the results and wrote the manuscript.

## Additional Information

**How to cite this article**: Brévault, T. *et al*. A seed mixture increases dominance of resistance to Bt cotton in *Helicoverpa zea*. *Sci. Rep.* **5**, 9807; doi: 10.1038/srep09807 (2015).

## Supplementary Material

Supporting Information

## Figures and Tables

**Table 1:** Simulated evolution of resistance to 78% Bt cotton and 22% non-Bt cotton in a seed mixture vs. blocks of Bt cotton and non-Bt cotton.

**Refuge type**	**Larval survival (%) in blocks of non-Bt cotton**	Number of generations to resistance*
Seed mixture	Not applicable	8
Block	35	16
Block	65	26
Block	95	36

^*^Resistance allele frequency ≥0.5.
