# Supplementary material for: A seed mixture increases dominance of resistance to Bt cotton in Helicoverpa zea
Source: Sci Rep. 2015 May 7;5:9807. doi: 10.1038/srep09807 (PMC4423431; doi:10.1038/srep09807)
Supplement: Supporting Information [file srep09807-s1.pdf]

A seed mixture increases dominance of resistance to Bt cotton in *Helicoverpa zea*

Thierry Brévault, Bruce E. Tabashnik & Yves Carrière

Supplementary Information

Supplementary Methods 1

Insects. We used two previously described strains of *H. zea*[1]: a field-derived strain from Georgia that was exposed to Bt toxins only in the field (GA), and a strain derived from GA that was selected in the laboratory with Cry1Ac in diet for nine generations (GA-R), as detailed below. We provided moths with cotton balls wetted with a 10% dilution of honey in water for feeding and cheesecloth for egg-laying. Eggs were harvested daily and larvae were reared on diet (Southland Products Inc.). Strains were maintained at 27 ± 1°C, 60 ± 10% relative humidity (RH), and 14 light (L):10 dark (D).

The GA strain originated from 180 larvae collected in July 2008 from Cry1Ab corn (*Zea mays* L.) hybrid DKC 6971 (MON810) near Tifton, Georgia, and was reared on diet without exposure to toxins. After two generations of laboratory rearing, we used a subset of insects from GA to start the GA-R strain. We selected GA-R for resistance to Cry1Ac during each of nine non-consecutive generations by exposing at least 1,000 GA-R neonates to Cry1Ac in diet. In each selected generation, only larvae that reached third instar after 7 d of feeding on diet treated with Cry1Ac were transferred to non-Bt diet and reared to pupation to continue the strain. The concentration (μg of Cry1Ac mL^{-1} diet) increased progressively from 10-20 in selected generations 1-3 to 100-1,000 in selected generations 4-9.

To evaluate survival on non-Bt and Cry1Ac cotton, we tested neonates (<24 h old) from GA, GA-R and their F_1 progeny obtained from reciprocal crosses conducted with a minimum of 30 mating pairs. Each neonate was individually transferred to a terminal leaf of a cotton plant in the greenhouse. F1 progeny from both types of reciprocal crosses (GA male × GA-R females and GA-R males × GA females) were pooled. No significant difference in survival to Cry1Ac cotton between the F1 progeny from reciprocal crosses was observed in an experiment conducted concurrently with this study[1].

Plants. We used cultivars of Bt cotton producing Cry1Ac (DP 448 B) and non-Bt cotton (DP 5415). Cotton plants were grown in a greenhouse at the University of Arizona in Tucson under drip irrigation in 20 L plastic pots (30 cm diameter) containing standard potting soil mixture (Sunshine soil mix 1, Sun Gro Horticulture, Canada). Three plants were planted in each pot. Supplementary fertilization was provided through the application of 20-20 fertilizer (Peters Professional general purpose for continuous feed programs, Marysville, OH) at 30, 45 and 60 days after planting. Climatic conditions prevailing during the experiments were 30 ± 3°C, 60 ± 15% RH, and photoperiod 14L:10D.

Experimental design. Each plant array had three pots placed 20 cm apart (Fig. S1 and S2), so that the plant density within an array was similar to that in farmers' fields. Experiments started at early fruiting, 55-68 days after planting. At this time, the leaves touched among plants within each array, facilitating larval movement between plants (Fig. S1).

For each of three experimental dates (i.e., temporal replicate), three spatial replicates were used. Each spatial replicate contained three arrays of Bt cotton and three arrays of the seed mixture, which were randomized within each spatial replicate (Fig. S2). Arrays within each spatial replicate were separated by sticky bands (Tanglefoot, Grand Rapids, MI) to prevent larvae from moving between arrays (Fig. S1). Thus, a total of 27 Bt cotton arrays and 27 seed mixture arrays were used in this experiment (3 temporal replicates × 3 spatial replicates × 3 arrays of either type), which provided a total of nine replicates for each insect type (i.e., GA, GA-R, and F1) on Bt cotton or seed mixture.

Data analyses. Supplementary Tables 1-3 detail results from statistical analyses. Supplementary Data 1-3 contain data used in analyses. In the ANOVA results summarized in Supplementary Tables 1 and 3, we estimated variance components using the REML method in JMP 9.0 (SAS Institute, Cary, NC). We evaluated dominance (h) in 100% Bt and seed mixture arrays for each of the nine spatial replicates in the experiment (Supplementary Table 1). The data for h from one spatial replicate (i.e., spatial replicate 6) were not included in the ANOVA, because survival on Bt cotton was 0% for all three genotypes, which precludes calculation of h; and survival was lower for GA-R than for GA and F1 in the seed mixture, which yields a negative estimate of h. In analyzing the distribution of larvae of Bt and non-Bt plants in seed mixtures (Table S2), we did not consider first instars because they were small and difficult to find on the plants.

Calculation of dominance (*h*) when some resistance alleles occur in a primarily susceptible strain.

The standard calculation of the dominance parameter *h* is:

(1) $h = (W_{rs} - W_{ss}) / (W_{rr} - W_{ss})$

where W_{ss}, W_{rs}, and W_{rr} are the fitnesses of the genotypes *ss* (susceptible homozygote), *rs* (heterozygote), and *rr* (resistant homozygote), respectively[2].

Here we estimated fitness for GA, GA-R and their F1 progeny as survival from neonate to pupa on either a block of Bt cotton or a seed mixture with 78% Bt cotton and 22% non-Bt cotton. If all the individuals in GA are *ss* and all in GA-R are *rr,* then all of their F1 progeny are *rs,* and estimation of *h* from equation (1) is straightforward. Here we evaluated the potential effects on *h* of resistance alleles in the GA strain, because this strain had been exposed to Bt toxins in the field and was not a pure susceptible strain[1].

The GA strain initially had 55-fold resistance to Cry1Ac in diet bioassays relative a laboratory susceptible strain[1]. Before the current study, GA-R had 560-fold resistance to Cry1Ac relative to a laboratory susceptible strain[1]. Although it is reasonable to assume that GA-R was fixed for resistance alleles (*rr*), GA was probably not fixed for alleles conferring susceptibility. Thus, we determined the effects on *h* of four frequencies of *r* alleles in GA: 0, 0.05, 0.10, and 0.20. We found that the increase in *h* in the seed mixture relative to the block of Bt cotton increased as the frequency of *r* alleles in the GA strain increased (Supplementary Table 4). The calculation of *h* when *r* alleles are present in GA (or any primarily susceptible strain) is detailed below and can be done with an Excel spreadsheet available from the authors. In general, the overestimation of *h* caused by *r* alleles in a putative susceptible strain increases as the true value of *h* decreases below 0.5 (i.e., resistance is more recessive) because recessive *r* alleles increase the survival of F1 much more than they increase the survival of the putative susceptible strain. Conversely, the underestimation of *h* caused by *r* alleles in a putative susceptible strain increases as the true value of *h* increases above 0.5 (i.e., resistance is more dominant) because dominant *r* alleles increase the survival of F1 less than they increase the survival of the putative susceptible strain.

Dominance (h) on blocks of Bt cotton. Mean survival on blocks of Bt cotton was 0% for GA, 5.6% for F1, and 12% for GA-R (Fig. 1) with n = 162 for each of the three strains (9 replicates of 18 larvae each per strain). The 0% survival of GA on blocks of Bt cotton

indicates that *r* alleles conferring survival on Bt cotton were not common, and that $W_{ss} = 0$ in this strain on blocks of Bt cotton. However, we cannot rule out the presence of rare *r* alleles in GA that would have inflated survival of F1 on blocks of Bt cotton.

We evaluated the effect of *r* alleles in GA on survival of F1 as follows:

(2) $W_{F1} = (W_{rs} \times a) + (W_{rr} \times b)$

where W_{F1} is the observed survival of F1 progeny, and *a* and *b* are the frequency of *rs* and *rr* in the F1 progeny, respectively.

With no *r* alleles in GA, $a = 1$, $b = 0$, and equation 2 simplifies to $W_{F1} = W_{rs}$. However, when *r* alleles are present in GA, we can rearrange equation 2 to yield W_{rs} as a function of the survival of F1 and the frequency of *rs* and *rr* in the F1 progeny:

(3) $W_{rs} = (W_{F1} - (W_{rr} \times b)) / a$

Because the F1 progeny were produced by crossing GA with GA-R (*rr*), we can calculate *a* and *b* as:

(4) $a = c + 0.5\text{d}$

(5) $b = e + 0.5\text{d}$

where *c*, *d*, and *e* are the frequency in GA of *ss, rs,* and *rr*, respectively.

We assumed Hardy-Weinberg equilibrium in GA and calculated each of the three genotype frequencies in GA (*c*, *d*, and *e*) from the assumed frequency of the *r* allele in GA: 0, 0.05, 0.1, or 0.2. We calculated *h* by using the value of W_{rs} from equation 3 in equation 1:

(6) $h = [(W_{F1} - (W_{rr} \times b)) / a] - W_{ss}] / [W_{rr} - W_{ss}]$

As noted above, in blocks of Bt cotton, $W_{ss} = 0$, which allows simplification of equation 6:

(7) $h = [(W_{F1} - (W_{rr} \times b)) / a] / W_{rr}$

With the *r* allele frequency in GA = 0, *a* and *b* = 0, and equation 7 simplifies to $h = W_{F1}/W_{rr} = 0.056/0.12 = 0.47$. With the *r* allele frequency in GA = 0.10, the Hardy-Weinberg principle yields $c = 0.81$, $d = 0.18$, and $e = 0.01$, equations 4 and 5 yield $a = 0.90$ and $b = 0.10$, equation 3 yields $W_{rs} = 0.05$, and equation 7 yields $h = 0.42$. In this case, nearly all of the *r* alleles in GA are in *rs*, *h* is less than 0.5, and the *r* alleles do not increase the survival of GA

as much as they increase the survival of F1. The *r* alleles inflate the survival of F1 more than the survival of GA because in F1 they occur at a substantial frequency in *rr* as well as in *rs.* So, the true value of *h* is overestimated if the *r* alleles in GA are ignored. Moreover, this overestimation of *h* in blocks of Bt cotton increases as the frequency of *r* alleles in GA increases (Supplementary Table 4).

Dominance (h) in a seed mixture. Mean survival in the seed mixture of Bt and non-Bt cotton was 6.2% for GA, 16.6% for F1, and 19.8% for GA-R (Fig. 1). In the seed mixture, with survival of GA > 0, the relationship between survival of GA (W_{GA}) and survival of *ss* (W_{ss}) is:

(8) $W_{GA} = (W_{ss} \times c) + (W_{rs} \times d) + (W_{rr} \times e)$

which can be rearranged as :

(9) $W_{ss} = (W_{GA} - (W_{rs} \times d) - (W_{rr} \times e)) / c$

With the *r* allele frequency in GA = 0, $c = 1$ and a, b, d and $e = 0$, $W_{ss} = W_{GA}$ and $W_{rs} = W_{F1}$. In this case, equation 1 can be written as $h = (W_{F1} - W_{ss})/(W_{rr} - W_{ss})$, which for the seed mixture is 0.104/0.136 = 0.76. As above for blocks of Bt cotton, an *r* allele frequency in GA = 0.10 yields $c = 0.81$, $d = 0.18$, $e = 0.01$, $a = 0.90$ and $b = 0.10$. In this case, equation 9 yields $W_{ss} = 0.037$, equation 3 yields $W_{rs} = 0.162$, and equation 1 yields $h = 0.78$ for the seed mixture. In the seed mixture, the *r* alleles are more dominant than in the Bt cotton blocks, and their presence in GA increases the survival of GA relatively more than they increase the survival of F1. In the seed mixture, because the *r* alleles in GA have a relatively large effect on survival of GA, the true value of *h* is underestimated if the *r* alleles in GA are ignored. This underestimation of *h* in the seed mixture increases as the frequency of *r* alleles in GA increases (Supplementary Table 4). Because ignoring *r* alleles in GA causes underestimation of *h* in the seed mixture and overestimation of *h* in blocks of Bt cotton, the true increase in *h* in the seed mixture relative to Bt cotton blocks is increasingly underestimated as the frequency of *r* alleles in GA increases (Supplementary Table 4).

References

1. Brévault, T., Heuberger, S., Zhang, M., Ellers-Kirk, C., Ni, X., Masson, L., Li, X., Tabashnik, B. E. & Carrière, Y. Potential shortfall of pyramided transgenic cotton for insect resistance management. *Proc. Natl. Acad. Sci. U. S. A.* **110**, 5806–5811 (2013).

2. Liu, Y.B. & Tabashnik, B.E. Inheritance of resistance to the *Bacillus thuringiensis* toxin Cry1C in the diamondback moth. Appl. Environ. Microbiol. **63**, 2218–2223 (1997).

Supplementary Table 1. Results of three-way ANOVA evaluating effects on dominance of resistance of plant configuration (seed mixture vs. 100% Bt cotton), temporal replicate, and spatial replicate nested within temporal replicate (random effect).

**Random effect**	**Variance component**	**Standard error**	**% of Total**
Spatial replicate [temporal replicate]	0.058	0.057	51.1
Residual	0.056	0.030	48.9
Total	0.114		100

**Fixed effects**	**F**	**Df**	***P***
Temporal replicate	0.81	2, 5	0.49
Plant configuration	5.7	1, 7	0.048

Supplementary Table 2. Results of logistic regression analysis comparing survival to pupation of GA, GA-R, and F1 in 100% Bt cotton and seed mixtures. Explanatory variables included in models were temporal replicate, spatial replicate nested within temporal replicate, and genotype.

**Effects**	**X^2**	**Df**	***P***
**100% Bt arrays**			
Temporal replicate	2.4	2	0.30
Spatial replicate [temporal replicate]	11.0	6	0.087
Genotype	27.9	2	< 0.0001
**Seed mixture arrays**			
Temporal replicate	0.65	2	0.72
Spatial replicate [temporal replicate]	8.2	6	0.22
Genotype	15.3	2	0.0005

Supplementary Table 3. Results of ANOVA comparing the percentage of larvae on non-Bt cotton plants in seed mixtures. The explanatory variables were temporal replicate, spatial replicate nested within temporal replicate (random effect), plant array nested within temporal and spatial replicate (random effect), genotype, instar, and the interaction between genotype and instar.

**Random effects**	**Variance component**	**Standard error**	**% of Total**
Spatial replicate [temporal replicate]	-77.5	49.7	-8.1
Array [Spat replicate, Temp replicate]	182.7	132.7	19.1
Residual	850.5	128.4	89.0
Total	955.8		100

**Fixed effects**	**F**	**Df**	***P***
Temporal replicate	8.3	2, 6.0	0.02
Genotype	0.89	2, 15.9	0.89
Instar	0.86	4, 89.7	0.86
Genotype × Instar	0.73	8, 89.4	0.73

Supplementary Table 4. Effects on dominance (*h*) of *r* alleles in the GA strain. If *r* alleles are present in GA, then the increase in *h* in seed mixture relative to block of Bt cotton calculated from observed fitness of the GA, F1 and GA-R strains (i.e., 0.29) is underestimated compared to the true increase in *h* (0.32, 0.36 and 0.45 for hypothetical *r* frequency of 0.05, 0.1 and 0.20 in GA, respectively).

***r* allele frequency in GA**	***h* in seed mix**	***h* in block of Bt cotton**	**Increase in *h* in seed mixture relative to block of Bt cotton**
**0.00**	**0.76**	**0.47**	**0.29**
**0.05**	**0.77**	**0.45**	**0.32**
**0.10**	**0.78**	**0.42**	**0.36**
**0.20**	**0.79**	**0.34**	**0.45**

Supplementary Data 1. Dominance of resistance in 100% Bt blocks and seed mixture arrays.

Temporal replicate	Spatial replicate	Mixture	*h*
1	1	Bt	0.67
1	1	Mix	1.3
1	2	Bt	0
1	2	Mix	0.5
1	3	Bt	0
1	3	Mix	0.33
2	4	Bt	0.67
2	4	Mix	0.67
2	5	Bt	0.5
2	5	Mix	0.6
2	6	Bt	-
2	6	Mix	-
3	7	Bt	0.5
3	7	Mix	1
3	8	Bt	1
3	8	Mix	0.67
3	9	Bt	0.5
3	9	Mix	1

Supplementary Data 2. Survival of genotypes in 100% Bt blocks and seed mixture arrays.

Temporal replicate	Spatial replicate	Genotype	Mixture	Survived	Total
1	1	GA-R	Bt	3	18
1	1	GA-R	Mix	5	18
1	1	F1	Bt	2	18
1	1	F1	Mix	6	18
1	1	GA	Bt	0	18
1	1	GA	Mix	2	18
1	2	GA-R	Bt	4	18
1	2	GA-R	Mix	4	18
1	2	F1	Bt	0	18
1	2	F1	Mix	2	18
1	2	GA	Bt	0	18
1	2	GA	Mix	0	18
1	3	GA-R	Bt	1	18
1	3	GA-R	Mix	4	18
1	3	F1	Bt	0	18
1	3	F1	Mix	2	18
1	3	GA	Bt	0	18
1	3	GA	Mix	1	18
2	4	GA-R	Bt	3	18
2	4	GA-R	Mix	4	18
2	4	F1	Bt	2	18
2	4	F1	Mix	3	18
2	4	GA	Bt	0	18
2	4	GA	Mix	1	18
2	5	GA-R	Bt	2	18
2	5	GA-R	Mix	5	18
2	5	F1	Bt	1	18
2	5	F1	Mix	3	18
2	5	GA	Bt	0	18
2	5	GA	Mix	0	18
2	6	GA-R	Bt	0	18
2	6	GA-R	Mix	1	18
2	6	F1	Bt	0	18
2	6	F1	Mix	3	18
2	6	GA	Bt	0	18
2	6	GA	Mix	2	18
3	7	GA-R	Bt	2	18
3	7	GA-R	Mix	4	18
3	7	F1	Bt	1	18
3	7	F1	Mix	4	18
3	7	GA	Bt	0	18
3	7	GA	Mix	3	18
3	8	GA-R	Bt	2	18
3	8	GA-R	Mix	3	18
3	8	F1	Bt	2	18

3	8	F1	Mix	2	18
3	8	GA	Bt	0	18
3	8	GA	Mix	0	18
3	9	GA-R	Bt	2	18
3	9	GA-R	Mix	2	18
3	9	F1	Bt	1	18
3	9	F1	Mix	2	18
3	9	GA	Bt	0	18
3	9	GA	Mix	1	18

Supplementary Data 3. Distribution of genotypes on Bt and non-Bt cotton in seed mixture arrays.

Temporal replicate	Spatial replicate	Array	Instar	Genotype	Larvae on non-Bt cotton	Total larvae observed	% larvae on non-Bt cotton
1	1	2	2	GA	2	6	33.3
1	1	3	2	F1	2	8	25
1	1	5	2	GA-R	1	4	25
1	1	2	3	GA	2	6	33.3
1	1	3	3	F1	6	11	54.5
1	1	5	3	GA-R	0	6	0
1	1	2	4	GA	1	2	50
1	1	3	4	F1	0	3	0
1	1	5	4	GA-R	0	4	0
1	1	2	5	GA	1	2	50
1	1	3	5	F1	6	8	75
1	1	5	5	GA-R	1	3	33.3
1	1	2	6	GA	2	5	40
1	1	3	6	F1	5	10	50
1	1	5	6	GA-R	1	10	10
1	2	1	2	F1	4	15	26.7
1	2	2	2	GA-R	3	10	30
1	2	4	2	GA	1	5	20
1	2	1	3	F1	1	4	25
1	2	2	3	GA-R	2	7	28.6
1	2	4	3	GA	2	4	50
1	2	1	4	F1	1	1	100
1	2	2	4	GA-R	0	1	0
1	2	4	4	GA	0	1	0
1	2	1	5	F1	1	2	50
1	2	2	5	GA-R	0	6	0
1	2	4	5	GA	-	-	-
1	2	1	6	F1	5	5	100
1	2	2	6	GA-R	0	10	0
1	2	4	6	GA	-	-	-
1	3	2	2	F1	1	6	16.7
1	3	3	2	GA	1	2	50
1	3	5	2	GA-R	1	4	25
1	3	2	3	F1	2	8	25
1	3	3	3	GA	1	1	100
1	3	5	3	GA-R	2	8	25
1	3	2	4	F1	0	3	0
1	3	3	4	GA	0	1	0
1	3	5	4	GA-R	1	3	33.3
1	3	2	5	F1	0	2	0
1	3	3	5	GA	-	-	-
1	3	5	5	GA-R	0	4	0
1	3	2	6	F1	0	5	0

1	3	3	6	GA	0	3	0
1	3	5	6	GA-R	0	11	0
2	4	1	2	F1	3	9	33.3
2	4	3	2	GA-R	4	8	50
2	4	4	2	GA	4	9	44.4
2	4	1	3	F1	1	4	25
2	4	3	3	GA-R	0	3	0
2	4	4	3	GA	1	3	33.3
2	4	1	4	F1	-	-	-
2	4	3	4	GA-R	1	5	20
2	4	4	4	GA	0	1	0
2	4	1	5	F1	0	5	0
2	4	3	5	GA-R	2	5	40
2	4	4	5	GA	0	1	0
2	4	1	6	F1	0	7	0
2	4	3	6	GA-R	3	9	33.3
2	4	4	6	GA	0	2	0
2	5	2	2	GA-R	1	5	20
2	5	4	2	F1	1	4	25
2	5	6	2	GA	4	7	57.1
2	5	2	3	GA-R	1	8	12.5
2	5	4	3	F1	0	2	0
2	5	6	3	GA	0	2	0
2	5	2	4	GA-R	3	8	37.5
2	5	4	4	F1	1	3	33.3
2	5	6	4	GA	0	3	0
2	5	2	5	GA-R	2	3	66.7
2	5	4	5	F1	1	2	50
2	5	6	5	GA	-	-	-
2	5	2	6	GA-R	5	14	35.7
2	5	4	6	F1	6	8	75
2	5	6	6	GA	-	-	-
2	6	2	2	GA-R	1	5	20
2	6	3	2	F1	3	6	50
2	6	5	2	GA	1	8	12.5
2	6	2	3	GA-R	-	-	-
2	6	3	3	F1	2	11	18.2
2	6	5	3	GA	2	3	66.7
2	6	2	4	GA-R	1	2	50
2	6	3	4	F1	1	3	33.3
2	6	5	4	GA	1	3	33.3
2	6	2	5	GA-R	0	1	0
2	6	3	5	F1	0	2	0
2	6	5	5	GA	1	3	33.3
2	6	2	6	GA-R	1	1	100
2	6	3	6	F1	0	8	0
2	6	5	6	GA	0	7	0
3	7	4	2	GA	3	7	42.9

3	7	5	2	GA-R	1	4	25
3	7	6	2	F1	1	4	25
3	7	4	3	GA	2	5	40
3	7	5	3	GA-R	1	1	100
3	7	6	3	F1	1	2	50
3	7	4	4	GA	5	5	100
3	7	5	4	GA-R	1	4	25
3	7	6	4	F1	1	4	25
3	7	4	5	GA	2	7	28.6
3	7	5	5	GA-R	3	3	100
3	7	6	5	F1	3	7	42.9
3	7	4	6	GA	1	4	25
3	7	5	6	GA-R	5	11	45.4
3	7	6	6	F1	3	6	50
3	8	2	2	GA-R	2	3	66.7
3	8	4	2	F1	0	1	0
3	8	6	2	GA	1	5	20
3	8	2	3	GA-R	3	4	75
3	8	4	3	F1	0	1	0
3	8	6	3	GA	1	2	50
3	8	2	4	GA-R	3	6	50
3	8	4	4	F1	1	4	25
3	8	6	4	GA	1	1	100
3	8	2	5	GA-R	2	5	40
3	8	4	5	F1	1	2	50
3	8	6	5	GA	2	2	100
3	8	2	6	GA-R	0	5	0
3	8	4	6	F1	4	4	100
3	8	6	6	GA	2	2	100
3	9	1	2	F1	1	2	50
3	9	2	2	GA-R	2	4	50
3	9	6	2	GA	0	2	0
3	9	1	3	F1	2	2	100
3	9	2	3	GA-R	2	4	50
3	9	6	3	GA	-	-	-
3	9	1	4	F1	2	2	100
3	9	2	4	GA-R	1	1	100
3	9	6	4	GA	0	2	0
3	9	1	5	F1	1	3	33.3
3	9	2	5	GA-R	0	2	0
3	9	6	5	GA	0	1	0
3	9	1	6	F1	2	4	50
3	9	2	6	GA-R	0	4	0
3	9	6	6	GA	0	2	0

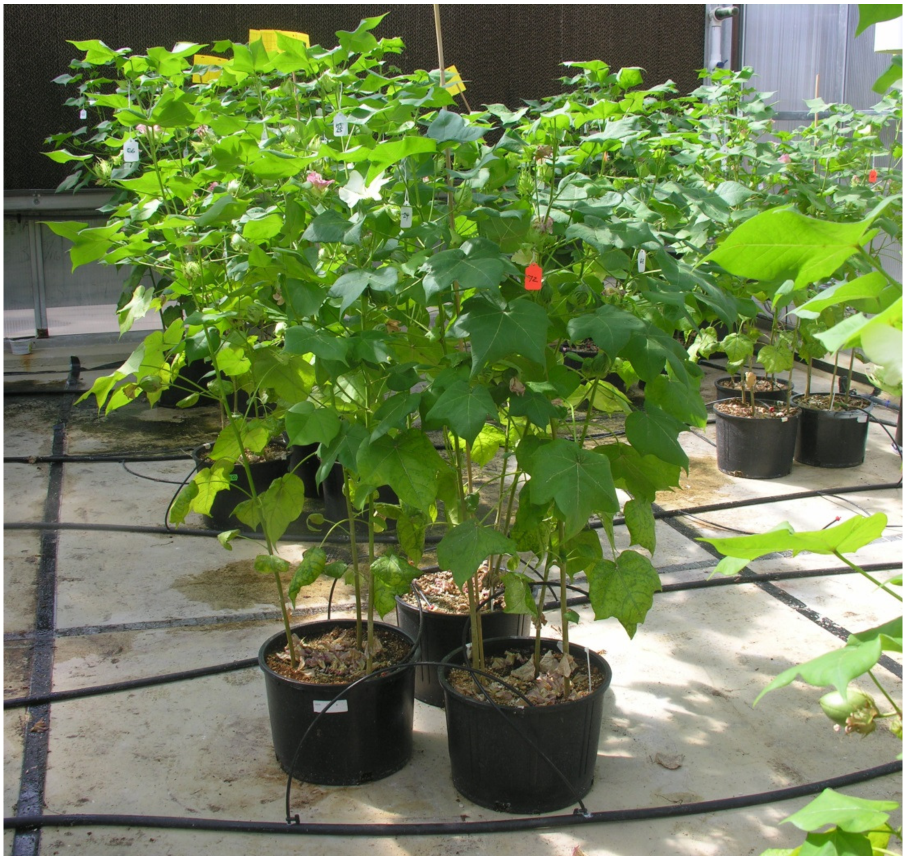

Supplementary Fig. 1. Each plant array had nine cotton plants; three pots with three cotton plants in each pot. Within each array, the leaves touched among plants, facilitating larval movement between plants.

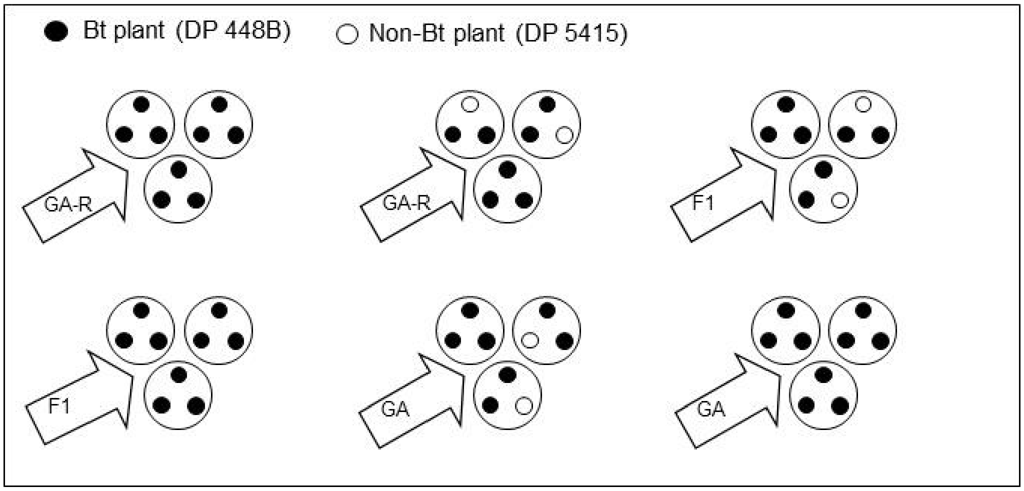

Supplementary Fig. 2. One spatial replicate of the experimental design. Each array had nine cotton plants. The arrays for 100% Bt blocks had nine Bt cotton plants and the seed mixture arrays had seven Bt cotton plants (78%) and two non-Bt cotton plants (22%). Plants within pots, pots within arrays, and arrays within each spatial replicate were distributed randomly and infested with neonates of the appropriate genotype.
